# COVID-19–Induced Fear in Infoveillance Studies: Pilot Meta-analysis Study of Preliminary Results

**DOI:** 10.2196/21156

**Published:** 2021-02-03

**Authors:** Styliani Geronikolou, George Chrousos

**Affiliations:** 1 University Research Institute of Maternal and Child Health and Precision Medicine National and Kapodistrian University of Athens Athens Greece

**Keywords:** COVID-19, social media, misinformation, infodemics, infodemiology, infoveillance, fear, meta-analysis

## Abstract

**Background:**

The World Health Organization named the phenomenon of misinformation spread through social media as an “*infodemic*” and recognized the need to curb it. Misinformation infodemics undermine not only population safety but also compliance to the suggestions and prophylactic measures recommended during pandemics.

**Objective:**

The aim of this pilot study is to review the impact of social media on general population fear in “*infoveillance*” studies during the COVID-19 pandemic.

**Methods:**

The PRISMA (Preferred Reporting Items for Systematic Reviews and Meta-Analyses) protocol was followed, and 6 out of 20 studies were retrieved, meta-analyzed, and had their findings presented in the form of a forest plot.

**Results:**

The summary random and significant event rate was 0.298 (95% CI 0.213-0.400), suggesting that social media–circulated misinformation related to COVID-19 triggered public fear and other psychological manifestations. These findings merit special attention by public health authorities.

**Conclusions:**

Infodemiology and infoveillance are valid tools in the hands of epidemiologists to help prevent dissemination of false information, which has potentially damaging effects.

## Introduction

The COVID-19 pandemic has raised health care, hospitalization, and research demands in an exponential manner. Apart from the burden of the confirmed cases and the high mortality rates, this pandemic has strained the public health systems of several countries. The World Health Organization (WHO) characterized this outbreak as a Public Health Emergency of International Concern [1,2]. In addition, the WHO identified potentially damaging misinformation spread through social media, or “*infodemics*,” and recognized the need to curb it [3]. Indeed, citizens from all over the world were exposed to a plethora of information and misinformation, especially through social media, while public health authorities wrestled to broadcast evidence-based important information. *Infodemics* undermine compliance to health authority suggestions and prophylactic measures, and hence, compromises population safety. Moreover, misinformation challenges self-respect, personal rights, and survival instincts, causing fear, anxiety, panic, depression, and unpredictable behaviors such as violence and suicidal thoughts in the general population.

A recent systematic review recognized an increasing trend in studying social media misinformation during and after epidemics [4]. Previous reviews have illustrated the psychological and physical distress in health care professionals due to COVID-19 [5] and previous infectious epidemics [6,7]; however, the general population’s fear and behavioral expressions are yet to be established. Massive fear may trigger unpredictable social processes and may result in posttraumatic stress disorder (PTSD) [8]. The attempt to collect and interpret data from social media may reveal the dominant stressors in the epidemic, as well as information on personal and business communications. “*Infodemiology*” is a rapidly growing research field that collects internet data for epidemiologic and other public health needs [9,10]. The aim of this pilot study is to review the impact of social media on the negative sentiments of the general population in published “*infoveillance*” studies.

## Methods

Databases such as MEDLINE and PUBMED (The National Library of Medicine) were searched using the keywords “infodemics COVID-19” or “fear due to COVID-19 social media misinformation” or “infodemiology and COVID-19” or “COVID-19 and social media impact on mental health.” The literature search was conducted in mid-May 2020. The articles meeting the eligibility criteria were evaluated by the PRISMA (Preferred Reporting Items for Systematic Reviews and Meta-Analyses) guidelines [11] (Multimedia Appendix 1).

The inclusion criteria were English language studies related to social media, fear, and infoveillance data retrieved from social media. Reviews, meta-analyses, and opinion articles were excluded from this analysis. Two of the authors (SG and GC) searched and screened articles, and agreed on their quality; the articles were scored using the Newcastle-Ottawa Scale for risk of bias evaluation (Multimedia Appendix 2). The Cohen kappa for interrater agreement was 90% (0.66) for the abstract selection but 96% for the full inclusion of the study. Any disagreement was addressed by mutual consensus.

The population targeted was social media users expressing fear (posts; P) because they had been exposed to misinformation during the first phase of the COVID-19 pandemic (E) in comparison to the total posts of the specific social media during the same period (C). The outcome (O; “events” or fear posts) were presented in effect sizes and calculated as event rates (p = events / total reference population; the proportion of patients and events in a group in which the “event” is observed). We further calculated:

Event Rate p = event / total **(1)**

logit (LogitEventRate = Log(p / (1 – p)) **(2)**

where LogitEventSE = Sqr(1 / (p * Total) + 1 / ((1 – p) * Total)) **(3)**

or EventRate = (e ^ LogitEventRate) / (e ^ LogitEventRate + 1) **(4)**

The probability of fear (f): f = ExpLogit / (1 + ExpLogit) **(5)**

In this analysis, we applied and presented the random effects model, which assumes that the data being analyzed are drawn from a hierarchy of different populations [12]. We calculated the heterogeneity with I^2^ [13,14] and τ^2^ [15,16]. All calculations were performed in R software (R Foundation for Statistical Computing). The results are presented with their 95% CIs, and in the summary results, 95% prediction intervals were also estimated with Higgins et al’s [17] formula. Lwin et al [18] did not report absolute patient numbers but daily proportions. Thus, we estimated these numbers by calculating the mean from the first figure of the relevant publication.

## Results

Of the 20 studies retrieved originally [1,3,5,18-34], only 6 met the inclusion criteria [18,20-24].

**Figure 1:**
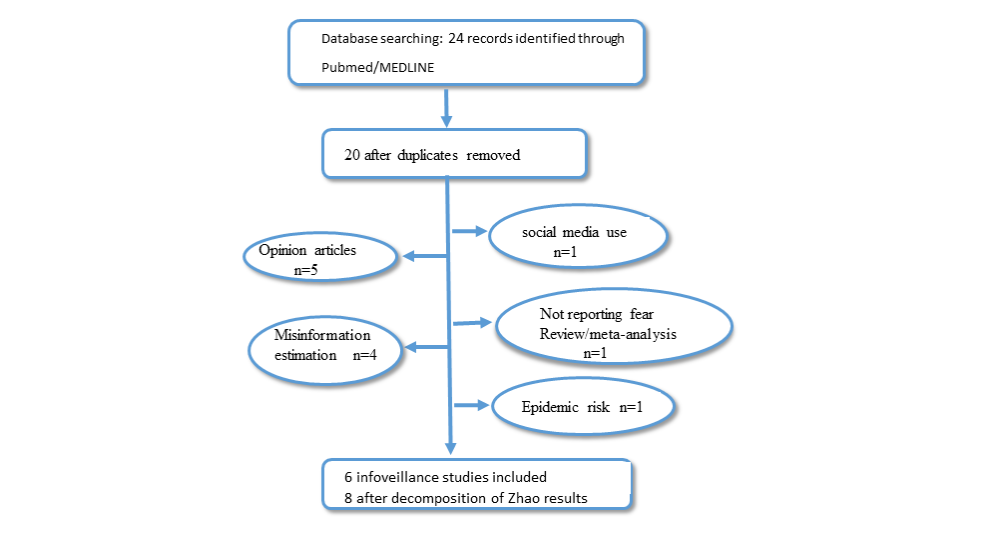
Flow chart.

One referred to the epidemic risks [34], 5 expressed opinions on infodemics [1,3,27,32,34], 1 counted social media use [25], 1 was a meta-analysis on depression and anxiety [5], and 4 estimated misinformation [19,26,29,31,33], and these were excluded from this study (Figure 1). As the Zhao et al [24] publication included three phases, we considered each phase as a separate study; thus, we summarized the results of 8 studies. We also included the Ahmad and Murad [20] and Gebbia et al [23] studies, even though they were actually surveys, because they were performed with data from Facebook and WhatsApp, respectively, and reported results on fear.

**Figure 2:**
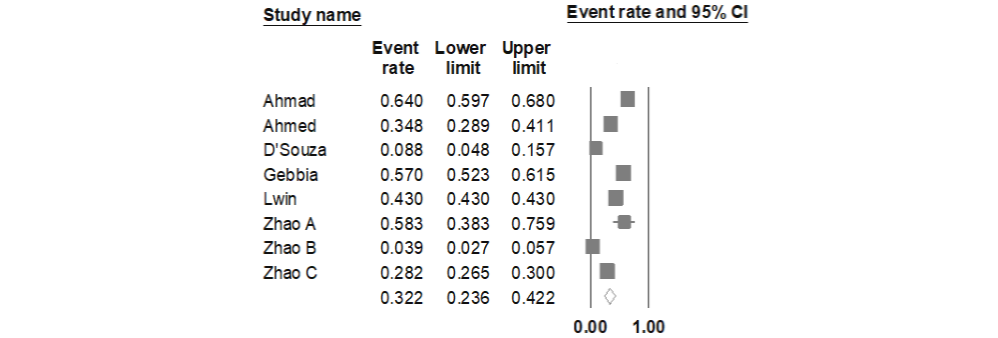
Forest plot of fear random event rates 95% CI due to Covid-19 surge retrieved by infodemics.

The studies included herein had processed over three million social media events (Facebook, YouTube, Twitter, WhatsApp, and similar versions in China) from over 170 countries, with messages expressed in seven languages (Table 1). In sum, out of 20,330,510 posts referring to COVID-19, 8,741,601 were retrieved that expressed fear. These studies were meta-analyzed using event rates, and their random effect is presented in Figure 2 and Table 2. The calculated LogitEventRate random effect was 0.746 (95% CI –1.176 to –0.315), while the summary odds was calculated as 0.475 (95% CI 0.3086 to 0.7295; 95% prediction intervals 0.1018 to 2.2119; Tables 2 and 3). The probability was 0.322. When we excluded the Gebbia et al [23] study, the random effect LogitEventRate was –0.907 (95% CI –1.387 to –0.428; 95% prediction intervals –2.6052 to 0.7903; SE 0.245; variance 0.06; probability 0.288; Tables 2 and 3). The Ahmad and Murad [20] study reported observations on Facebook (82.6%) and other social media sources; the observations were reported unstratified, and the results were presented as Facebook results.

**Table 1:** Studies characteristics.

Study	Age (years)	Gender (male/female), n	Total messages screened, n	Messages expressing fear, n	Social media
Ahmad and Murad (2020) [20]	18-35: 65.1%; >51: 6%	222/336	516	330	Facebook^a^
Ahmed et al (2020) [21]	—^b^	—	233	81	Twitter
D’Souza et al (2020) [22]	—	—	113	10	YouTube
Gebbia et al (2020) [23]	Range 34-90	190/252	446	254	WhatsApp
Lwin et al (2020) [18]	—	—	20,325,929	8,740,150	Twitter
Zhao et al (2020) [24], part A	Range 18-41	—	24	14	Sina microblog
Zhao et al (2020) [24], part B	Range 18-41	—	639	25	Sina microblog
Zhao et al (2020) [24], part C	Range 18-41	—	2610	737	Sina microblog
Total	N/A^c^	N/A	20,330,510	8,741,601	N/A

^a^82.6% of the observed messages came from Facebook.

^b^Data was not available.

^c^N/A: not applicable.

Sexual dimorphism was reported in 2 studies [20,23] in which women circulated more fear- inducing misleading posts. The methodology of the remaining studies did not include any relevant calculations, so the gender prevalence could not be taken into account.

The social media type probabilities are listed in Table 4. Of those, the Twitter-induced fear probability, as well as the overall probability, might be considered most credible (including many countries, ethnicities, and languages).

**Table 2:** Meta-analysis results.

Study	Event rate (95% CI)	Logit (95% CI)	SE	Variance	Weight random	Residual random (event rate)
Ahmad and Murad (2020) [20]	0.64 (0.597 to 0.680)	0.57 (0.394 to 0.753)	0.0917	0.008	13.53	2.38
Ahmed et al (2020) [21]	0.348 (0.289 to 0.411)	–0.63 (–0.899 to –0.36)	0.1376	0.019	13.14	0.21
D’Souza et al (2020) [22]	0.088 (0.048 to 0.157)	–2.33 (–2.981 to –1.683)	0.3312	0.110	10.53	–2.48
Gebbia et al (2020) [23]	0.57 (0.523 to 0.615)	0.28 (0.092 to 0.467)	0.0956	0.009	13.5	1.85
Lwin et al (2020) [18]	0.43 (0.43 to 0.43)	–0.28 (–0.283 to –0.28)	0.0004	0.000	13.86	0.85
Zhao et al (2020) [24], part A	0.583 (0.383 to 0.759)	0.34 (–0.475 to 1.15)	0.4140	0.171	9.28	1.58
Zhao et al (2020) [24], part B	0.039 (0.027 to 0.057)	–3.20 (–3.6 to –2.8)	0.2040	0.042	12.37	–4.2
Zhao et al (2020) [24], et al	0.282 (0.265 to 0.300)	–0.93 (–1.018 to –0.85)	0.0435	0.002	13.78	0.34
Random effect	0.322 (0.236 to 0.422)	–0.75 (–1.176 to –0.315)	0.219	0.048	N/A^a^	N/A
Random effect without Gebbia et al [23] study	0.288 (0.200 to 0.395)	–0.907 (–1.39 to –0.428)	0.245	0.06	N/A	N/A

^a^N/A: not applicable.

**Table 3:** Prediction intervals and probability of fear random effect in all studies and when Gebbia study is not considered.

Studies	Random effect logit (95% prediction intervals)	Probability
All studies	–0.7455 (–2.2849 to 0.7939)	0.322
Gebbia et al [23] study excluded	0.9075 (–2.6052 to 0.7903)	0.288

**Table 4:** Probability of fear effect for each social media.

Social media type	Studies, n	Total reference population, n	Country	Logit event rate	Exp	Probability
Twitter	2	20,326,162	>170 countries	0.428	0.651811	0.288
WhatsApp	1	446	Italy	0.28	1.32313	0.57
Facebook	1	516	Iraqi Kurdistan	0.573346	1.774194	0.64
YouTube	1	113	US	–2.33214	0.097087	0.088
Sina microblog	3	3273	China	–1.283	0.277204	0.217

## Discussion

Epidemics have caused burden on humankind since antiquity; past communities experienced shock that has been reflected in art, literature, massive population transitions, political turmoil, and changes in governance. Myths and legends evolved while people tried to deal with the unknown, the unpredictable, and the unexpected. Interpretations included, among others, divine interventions or punishment, conspiracy theories, religious fanaticism, racism, and scapegoating. Sparsity of data, especially in the beginning of an epidemic, facilitates misinformation spreading, and once this is initiated, “it is difficult to argue with reason” [35]. Interestingly, a recent psychology study established that “illusory pattern perceptions is a central cognitive function accounting for conspiracy theories and irrational beliefs” [36].

At the start of the current pandemic, the new coronavirus produced a broad clinical entity with an unpredictable natural history and uncertain treatment. The uncertainty caused feelings of fear, anxiety, and even depression, developing under an unexpected surge of serious morbidity and mortality [5,25,37,38].

These days, social media are a sine qua non for personal communications, business advertising, and updates [39]. During the pandemic, social media were used to empower the population and support public health measures. Yet, public health officials and academic researchers were alarmed by the size and spread of community confusion, frequently in response to “fake news” [21,25,27,40-42]. Thus, many nations were exposed to numerous misinformative communications regarding the origin of the epidemic (conspiracy theories, 5G antennas, etc), its transmission route (Asian neighbors, zoonotic or airborne transmission), the appropriate prophylactic measures (the herd immunity or isolation dilemma, vitamin and supplement effectiveness, etc), the treatment effectiveness (ibuprofen, hydroxychloroquine, etc), drug synergy (use of angiotensin-converting enzyme inhibitors, sartans), the vaccines expected (ineffective or even lethal), and the socioeconomic consequences (famine, unemployment). The scale of misinformation varied depending on the various political, religious, and cultural particularities of nations; however, the aforementioned issues were predominant in most countries. These characteristics influenced the between all and within Twitter studies’ variance in our study.

Fear is an emotion that is caused by personal and societal threats and uncertainty (like the COVID-19 surge), while anger may originate from uncertainties caused by other persons [43]. Other negative emotions such as anxiety and depression are intertwined, individuality dependent, and have been evaluated in a previous meta-analysis [5]. Fear motivates unpredicted behaviors and merits attention in public health planning. Moreover, it was previously shown that indirect exposure to mass trauma through the media can accelerate the clinical manifestations of PTSD [8]. For all the previously mentioned reasons, we concentrated on fear in this meta-analysis.

Our study shows that the general population’s fear was significantly dominant for one-third of the population due to COVID-19–related misinformation (Tables 2 and 3, and Figure 2). The effect was random (considering heterogeneity between and within studies) and of robust magnitude. Even when we excluded one study, the magnitude of the effect persisted, revealing that a considerable part of the population was negatively influenced by misinformation. More importantly, it was established recently that “tweet quality (misinformation vs. correct information) did not differ based on the number of likes or retweets, indicating that misinformation is as likely to spread and engage users as is the truth” [28]. Thus, the 5G conspiracy was spread through Twitter [21]. Zhao et al [24] reported that negative emotions decreased over time not only by habituation but also by the progress of scientific research, physical distancing, and the effectiveness of health care. The same was implied by Li et al [26], who studied 115,299 posts in 39 days but did not give numbers and was, thus, excluded from our analysis [26].

The importance and risk of communicating emotions through social media have been verified experimentally [44,45] and based on real data [27] and the history of other recent epidemics [2,46-48]. Comparing the summarized random size effect of fear p_f_ with p_i_ (insomnia relevant), p_a_ (anxiety relevant), and p_d_ (depression relevant) as reported by Pappa et al [5], we see that (p_f_=p_i_>p_d_=p_a_). The dominant effect of fear was similar to that causing insomnia but greater than that related to anxiety or depression. This is underlined by fear’s nature; it is a primal emotion linked to survival, which may lead to complex feelings and moods such as anxiety and depressive manifestations or even clinical anxiety and depression.

The sexual dimorphism reported in two studies is indicative but cannot be assumed representative, as these specific studies were specific to ethnicity and had a small sample size. This observation may be explained from the fact that women tend to worry and distress by potential threats [49-51] and misleading information on potential risks in social media.

Our pilot study shows that the probability of social media users to develop fear due to misinformation is 32.2% (Table 3). The probability of fear varies upon the media used and the ethnicity and culture. Not including the WhatsApp cohort (Gebbia et al [23] study) that was targeted to a COVID-19 high risk group (patients with cancer), the fear effect probability decreased to 28.8% (Table 3). This phenomenon is reasonable considering that patient groups are physically more vulnerable to the virus and, perhaps, mentally more sensitive to any information, particularly misinformation. The observed decrease, however, is quite small at 3.4%.

The prediction intervals calculated indicated that effects of future studies might fall on the same side of the null and perhaps on both sides if the Gebbia et al [23] study is excluded. The prediction intervals “naturally account for heterogeneity” according to Higgins et al [17]; however, these intervals were criticized for their validity in small meta-analyses (including those with <20 studies) [52,53]. The heterogeneity in this meta-analysis was vast and persisted even when we excluded confounding studies, extreme-sized studies, or groups of studies (Table 5). It may be attributed to the small size of the summarized studies or to multicultural profiling. Yet, this meta-analysis is of value because its preliminary results and “difficulties” may guide future analyses on more studies to investigate group differences in social media type or culture homogeneous populations.

This study has to be viewed under its limitations: its pilot character; the time and period of conductance; the prematurity of the findings; the diversity of social media type surveyed; the multiethnicity, multicultural, and multi-language extracted data; and the unavailability of culture, age, gender, and education data in the retrieved studies.

Future cohort studies should better include more details on demographic, culture, and language data for more precise epidemiologic analyses, extracting targeted public health directions.

In conclusion, fear probability due to circulating misleading information was 32.2% for the general population, while when patient groups were excluded, fear probability diminished by 3.4%. Ethnicity and the social media type seem to be the main moderators of fear. Infodemiology and infoveillance may provide insight in epidemiologic research and contribute to the efficacy of public health measures. More importantly, our study suggests that public health officials must meet the challenge of curbing misinformation on the disease and its effects so as to protect their own credibility and effectiveness.

**Table 5:** Intrinsic heterogeneity in each included study or social media type population.

Study	Social media type	I^2^	τ^2^
Ahmad and Murad (2020) [20]	Facebook	0.00	0.00
Ahmed et al (2020) [21]	Twitter	0.00	0.00
Lwin et al (2020) [18]	Twitter	0.00	0.00
Ahmed et al [21] and Lwin et al [18]	Twitter	84.35	0.051
D’Souza et al (2020) [22]	YouTube	0.00	0.00
Gebbia et al (2020) [23]	WhatsApp	0.00	0.00
Zhao et al (2020) [24], part A	Sina microblog	0.00	0.00
Zhao et al (2020) [24], Part B	Sina microblog	0.00	0.00
Zhao et al (2020) [24], Part C	Sina microblog	0.00	0.00
Zhao et al [24], parts A, B, and C	Sina microblog	98.45	2.228
All studies	Combined social media	98.828	0.348
Gebbia et al [23] study excluded	WhatsApp excluded	98.934	0.376

## Appendix

Multimedia Appendix 1 The PRISMA Protocol.

Multimedia Appendix 2 Newcastle-Ottawa Scale for risk of bias.

## Abbreviations

PRISMA: Preferred Reporting Items for Systematic Reviews and Meta-Analyses
PTSD: posttraumatic stress disorder
WHO: World Health Organization
