# Supplementary material for: COVID-19–Induced Fear in Infoveillance Studies: Pilot Meta-analysis Study of Preliminary Results
Source: JMIR Form Res. 2021 Feb 3;5(2):e21156. doi: 10.2196/21156 (PMC7860927; doi:10.2196/21156)
Supplement: Multimedia Appendix 2 [file formative_v5i2e21156_app2.pdf]

Item /Studies	Ahamed	Ahmed	D' Souza	Gebbia	Lwin	Zhao	Zhao	Zhao
Representativeness of the exposed cohort	✩★	✩★	✩★	✩★	✩★	✩★	✩★	✩★
Selection of the non-exposed cohort	✩★	★✩	★✩	★✩	★✩	★✩	★✩	★✩
Ascertainment of exposure	✩★	✩★	✩★	✩★	✩★	✩★	✩★	✩★
Demonstration that outcome of interest was not present at start of study						✩★	✩★	✩★
Comparability	✩★			✩★	★✩			
Assessment of outcome	✩★	✩★	✩★	✩★	✩★	✩	✩★	✩★
Was follow-up long enough for outcomes to occur				★	★	★✩	★ ★✩	★ ★✩
Adequacy of follow-up of cohorts						★✩	★✩	★✩

Researcher SG ★ Researcher GC ✩, n/a: not applicable
